# Subarachnoid versus General Anesthesia in Penile Prosthetic Implantation: Outcomes Analyses

**DOI:** 10.1155/2012/696752

**Published:** 2012-08-15

**Authors:** Gerard D. Henry, Antonino Saccà, Elizabeth Eisenhart, Mario A. Cleves, Andrew C. Kramer

**Affiliations:** ^1^Department of Urology, Regional Urology, 255 Bert Kouns - Industrial Loop, Shreveport, LA 71106, USA; ^2^Department of Urology, Vita-Salute San Raffaele University, Milan, Italy; ^3^Department of Biostatistics, University of Arkansas for Medical Sciences, Little Rock, AR 72205, USA; ^4^Department of Urology, University of Maryland School of Medicine, Baltimore, MD 21201, USA

## Abstract

The leading patient complaint during the perioperative period for penile prosthesis implantation is postoperative pain, while emesis and urticaria also affect the procedure's perceived success. In analyzing surgical outcomes, assessment of the anesthetic for postoperative pain and side effects should be included. This paper retrospectively reviews 90 consecutive, primary inflatable penile prosthetic operations performed by a single surgeon at one private medical center. Fifty-seven patients were included in final analysis. Patients who had more than one procedure that day or who used chronic pain medication were excluded. The type and amount of each drug used for each respective side effect (within the first 24 hours after procedure) were compared to determine relative benefit. Twenty patients received general anesthesia (denoted herein as “GA”) and 37 received spinal (or also known as subarachnoid) anesthesia (denoted herein as “SA”). Patients receiving GA had significantly greater (*P* < 0.0001) occurrence and amount of intravenous pain treatment than those receiving SA. Patients with SA required less intravenous pain medication and less treatment for nausea/emesis.

## 1. Introduction

Penile prosthetic surgery has undergone significant changes since its introduction in the 1970's and it is now considered a safe and effective method of treating end organ failure impotence. As established by Pearman, an early leader in research in the field of surgical erectile dysfunction, impotence is defined as the inability to gain or maintain an erection sufficient to sustain satisfactory intercourse due to pathology or deformation of the penis [1]. The history of modern surgical treatment for erectile dysfunction began with the development of the inflatable penile prosthesis by Scott in the 1970s [1]. The popularity of inflatable penile prosthetics has increased since, and as the early designs yielded high failure rates, multiple revisions to the design and material have taken place. Current penile prosthetic implantation procedures prove to be both reliable and durable, with approximately 18,000 devices implanted annually worldwide [2]. With increased social awareness regarding erectile dysfunction, it appears there will be significant increases in penile prosthetic implantations in the future.

In the past, postoperative complications of penile prosthetic surgery included, but were not limited to, infection, mechanical failure, device migration, sizing issues, and patient dissatisfaction. As penile prosthesis surgery remains elective, patient satisfaction is of paramount importance. In the immediate postoperative period, patients judge the success or failure of their implant surgery, in part, by the extent of postoperative pain. Prior studies have shown that patients have disparate views of their surgical experience which can be attributed to the type of anesthetic administered [3–13]. In an effort to resolve this disparity, we performed an outcome analysis of general anesthesia versus subarachnoid anesthesia to evaluate side effects of penile prosthesis implantation in the immediate postoperative period. Many of these data endpoints are being reported for the first time in the literature as they pertain to surgical treatment of erectile dysfunction/penile prosthesis implantation. 

We evaluated immediate postoperative pain, urticaria, and nausea/emesis between the two major types of anesthesia currently used. Our hypothesis was that due to the drastic evolution of penile prosthetic surgery, coupled with numerous technical improvements, the necessity for GA should be lessened. We contend that not only have these advancements lessened the necessity for GA they have also lessened the status of GA as the preferable anesthetic. It is our opinion that SA may provide improved analgesia for this short procedure while sparing patients the systemic effects of GA. 

## 2. Materials and Methods

A retrospective study was conducted by reviewing medical records for 90 consecutive penile prosthetic implantation procedures which were performed by one surgeon at a private institution. There was no standard anesthetic in existence at this center. All patients provided written, informed consent as approved by the local hospital institutional review board.

We determined *a priori* to exclude all cases that included more than one procedure during the operating room time. In addition, age, race, etiology of impotence, and presence of chronic pain medication usage were noted and evaluated. If a patient had been treated for chronic pain with medication, his records were excluded from the study. Data was collected for only the first 24 hours post-procedure as all patients stayed overnight under observation status. All patients had standardized orders for postoperative nursing care for medical treatment of their pain, nausea, and urticaria. The standard order for urticaria was diphenhydramine (0.5 mg/kg, maximum dose 50 mg). For nausea/emesis in the postanesthesia care unit, the order was ondansetron (0.1 mg/kg, maximum dose 4 mg), whereas on the inpatient ward, the standard order was promethazine (25–50 mg). 

For pain in the after anesthesia care unit, with a visual analog scale (VAS) of 4 or greater on a scale of 0 to 10, the standardized order was for hydromorphone (0.5 mg IV, titrated to 2 mg). For the treatment of pain on the inpatient ward, a VAS was used to give oxycodone and morphine as part of the standardized nursing orders.

Data were collected based on each variable for either GA or SA. The side effects observed included urticaria, emesis, and pain. The variables included: operation time, anesthetic time, and the administration of oxycodone, hydromorphone, morphine, promethazine, ondansetron, and diphenhydramine. The medication and dosage used to treat each side effect were recorded in milligrams and the time was recorded in minutes during the postoperative period. Each variable for each type of anesthetic was placed in a spreadsheet and the data were analyzed using Wilcoxon's rank-sum (Mann-Whitney *U*) test and Fisher's exact test.

## 3. Results 

Fifty-seven patients who underwent penile prosthetic implantations were included in the study: 20 surgeries were performed under GA and 37 were performed under SA. There was no significant difference in age (63.1 GA versus 60.8 SA), race, or etiology of impotence (23% diabetic GA versus 31% diabetic SA) between the GA and SA groups.

To determine the efficacy of each type of anesthetic, both operative and anesthesia time were measured. The GA mean anesthesia time was 91.7 minutes, with a standard deviation of 15.82 minutes. The SA cohort mean anesthesia time was 85.3 minutes, with a standard deviation of 15.7 minutes. The GA mean operative time was 35.4 minutes with a standard deviation of 7.4 minutes. Finally, the SA population had a mean operative time of 35.9 minutes with a standard deviation of 8.2 minutes. There were no differences between groups for anesthesia time or operative time (*P* = 0.2991 and *P* = 0.9399, resp.).

Comparisons were made as to whether the patient was treated for nausea/emesis and the specific treatment used for it (medication and dose). The two medications evaluated were promethazine and ondansetron (amount in milligrams). Within the GA sample, the mean doses of promethazine and ondansetron administered were 6.25 ± 13.1 mg and 0.2 ± 0.89 mg, respectively. Within the SA sample, the mean doses of promethazine and ondansetron administered were 7.77 ± 9.48 and 0.11 ± 0.66, respectively. There was a significant difference in whether or not the patient was treated for nausea/emesis with promethazine and ondansetron between GA and SA (*P* = 0.0489).

To determine whether or not patients experienced urticaria after SA or GA in penile prosthetic implant procedures, the dose (in milligrams) of diphenhydramine was compared. None of the 20 patients receiving GA were treated for urticaria, whereas 14% of the patients receiving SA were treated for urticaria, with 4.73 ± 12.95 mg of diphenhydramine. These results did not reach statistical significance (*P* = 0.1041). 

**Figure 1:**
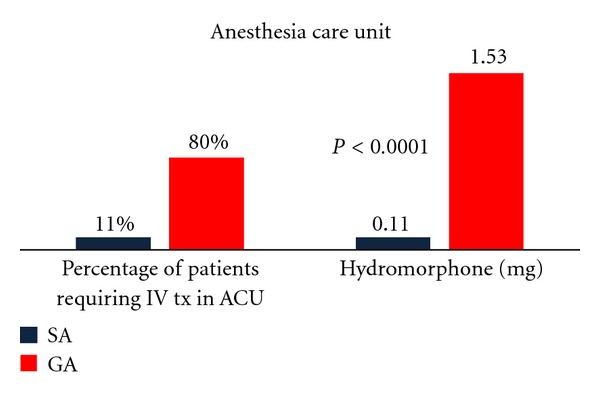
Percentage of patients requiring IV Hydromorphone and the dosage amount received by patients in the recovery room: spinal anesthesia in black versus general anesthesia in red.

**Figure 2:**
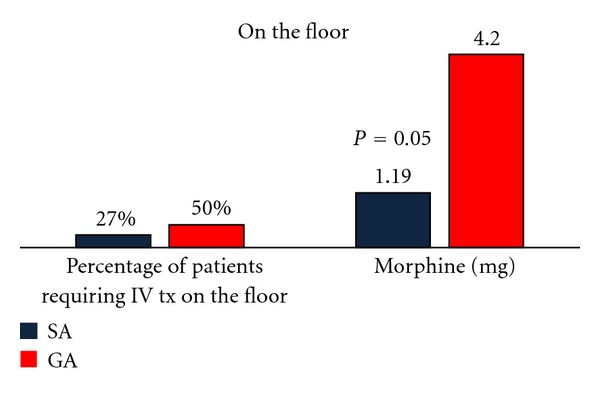
Percentage of patients requiring IV morphine and the dosage amount received by patients on the floor in the first 24 hours after surgery: spinal anesthesia in black versus general anesthesia in red.

In evaluating whether GA or SA benefited the patient in terms of postoperative pain, the doses of oxycodone, hydrocodone, morphine, and hydromorphone were compared. Data were analyzed by the number of tablets ingested and/or amount of intravenous medications administered. Hydromorphone was administered in the post-anesthesia care unit and morphine was administered while under observation on the inpatient ward. The dosages of oxycodone and hydrocodone were consistent (see Figures 1 and 2).There was no statistically significant difference at a 95% confidence interval (*P* = 0.4805) between anesthesia groups observed for pain which required treatment with oral medications.

The amount of intravenous pain medications administered was compared between GA or SA group with hydromorphone and morphine recorded in milligrams. The GA group required a significantly greater dosage of intravenous treatment for pain, in both the post-anesthesia care unit (hydromorphone) (*P* < 0.0001) and while under observation (morphine) (*P* = 0.05) on the inpatient ward (Figures 1 and 2). Additionally, there was a significant difference as to whether or not the patients required treatment with intravenous pain medications (*P* < 0.0001). 

Adding patients to each group would add credence to the study conclusions. Additionally, anesthesia varies among medical institutions. It remains unclear how these results might be affected by anesthesiologist experience in administering spinal and general anesthetic. It should also be noted that these results were taken from a private institution as opposed to a teaching hospital where SA administration can take considerably longer than GA administration. 

## 4. Discussion

The major complaint of patients undergoing penile prosthesis surgery in the perioperative period is surgical pain [14]. A review of the literature revealed no prior published papers addressing this complaint among patients undergoing an elective procedure where patient satisfaction remains of paramount importance. The overall purpose of this study was to explore the administration of GA and SA in primary penile prosthetic implantation procedures and determine whether there is any benefit of one anesthetic over the other. Previous studies have compared GA versus SA in terms of intraoperative and postoperative outcomes [15–24], with varying conclusions. Our study is the first to compare outcomes in penile prosthesis surgery; comparisons to existing literature possess limitations due to differences in the procedures which are performed.

Although there was no significant difference in operative or anesthetic time, a difference was discovered regarding need for intravenous pain medications and treatment of nausea/emesis. Of note, the study was done at a private hospital, so the anesthetic time could vary more at an academic setting with students/residents learning SA techniques. The treatment of urticaria trended toward significance, with a greater occurrence with the SA group. While comparing GA and SA in outpatient urological surgery, Erhan et al. [15] found no statistical differences in terms of urticaria (only 2 patients in the SA group) and their results were concordant with ours concerning the use of analgesia in GA group [15]. 

The question of whether regional anesthesia is better than general anesthesia has been debated since the inception of spinal anesthesia at the turn of the 20th century [16]. Regarding its efficacy, we did not find a significant difference between GA and SA in primary penile prosthetic implantations as measured by operative and anesthesia time. Many studies, including a large number of randomized control trials which assessed the effects of neuraxial anesthesia and analgesia on surgical outcome, have shown specific benefits of regional anesthesia. These benefits have included acceptable postoperative pain management, reduced thromboembolism, decrease in blood loss, and favorable postoperative effects on bowel motility. However, these studies have not consistently shown a difference in the realm of serious, life-threatening morbidity or mortality [16]. 

One obstacle in proving our hypothesis is that in most studies addressing the efficacy of SA compared to GA the differences in morbidity and mortality between any anesthetic techniques may be subtle. As a result, very large studies involving a multitude of patients would be required to power a comparison study [16]. A meta-analysis submitted by the University of Auckland in New Zealand, in which regional anesthesia was compared to general anesthesia, included 142 separate trials with a total of 9,553 patients. The regional anesthesia groups presented decreased overall mortality in the first month after surgery by approximately 30% (roughly one less death per 100 patients). Moreover they presented reduced incidence of deep vein thrombosis, pulmonary embolism, myocardial infarction, pneumonia, respiratory depression, need for blood transfusion, and post-operative renal failure. The researchers concluded that regional anesthesia is better than general anesthesia with regards to mortality and serious (but not fatal) morbidity [17].

For the benefit of reduced nausea/emesis after GA or SA, our study found a notable difference. Despite advances made in anesthesia, postoperative nausea and vomiting remains a dreaded anesthetic concern [18]. Antiemetic prophylaxis is effective, albeit expensive and is currently not recommended when there is little expectation of postoperative nausea and vomiting [18]. Nausea with emesis in the postoperative period is multifactorial and requires a review of multiple patient risk factors, including pre- and post-operative variables [19]. Some issues that increase the risk of postoperative nausea include female sex, obesity, prior history of anesthetic nausea, anxiety, opioid administration, use of volatile anesthetic agents, increased duration of surgical procedure, dehydration, and hypoxia [19]. Proper risk stratification may be beneficial in identifying those at risk in order to administer prophylactic antiemetics.

 A publication from Segovia, Spain, revealed significant reduction in intra-operative and postoperative nausea and vomiting in patients undergoing Cesarean section who received ondansetron or metoclopramide as compared with placebo [20]. Another study of men undergoing outpatient surgery cites that postoperative nausea and vomiting is perceived to be as debilitating as the consequences of surgery. Ondansetron (4 mg IV) given prophylactically was shown to be effective in the prevention of postoperative nausea and vomiting in the initial 24 hours [20].

Concerning urticaria, though insignificant, there was a trend toward a significant result demonstrating that SA recipients were more likely to experience urticaria. Although severe allergic reactions to medications in any setting are rare, certain patients do have increased sensitivity to injected agents. The importance of a thorough patient history is paramount whenever administering anesthesia. For patients with prior anaphylactic reactions to anesthetic agents, skin prick testing is available and may be used to identify agents responsible for the reaction in an effort to prevent peri-operative anaphylaxis and urticaria [21].

For postoperative pain control, there was a significantly lower requirement for IV pain medications among SA group patients yet there was no significant difference between SA and GA with respect to the administration of oral pain medications. Other studies have also shown that SA can result in less postoperative pain as compared with GA. A pilot study in urological practice was done by Salonia et al., evaluating postoperative outcomes among patients undergoing radical retropubic prostatectomy. In this study, patients with clinically localized prostate cancer were randomized into either a GA or SA group. SA resulted in decreased postoperative pain and faster postoperative recovery and proved to be a safe and effective alternative to GA in this study [22]. In a related clinical trial, researchers from Italy also compared different types of anesthesia for patients undergoing prostatectomy. Although this trial showed no significant improvement in operative conditions, it was found that arterial oxygen levels, gastrointestinal motility, and ambulation were superior in patients given SA. Furthermore, the patients that received SA had less postoperative pain [23]. A study by Gonano et al. compared the efficacy of GA versus SA in patients undergoing total hip or knee replacements. This study revealed SA to be the more effective anesthetic secondary to lower cost for anesthesia and with no appreciable difference in total anesthesia-related times. Additionally, the patients in the SA group reported lower postoperative pain scores in the post-anesthesia care unit [24].

This study is limited by small sample size and inconsistent anesthesia protocols. In addition, the study is limited in that the time spent in PACU was not standardized and that these results may not translate to outpatient (same day surgery) results. In the author's practice, SA is preferred for overnight observation but is not practical for outpatient (same day surgery) recovery time. The administration of spinal anesthetics, and to a lesser extent general anesthesia, may vary from case to case. Future research should include a prospective, randomized study in which the variables for each type of anesthetic can be controlled. Ideally, we envisage the possibility of a standard general anesthesia protocol for each patient. There might also be a standard protocol for SA to determine whether Duramorph had been administered intravenously or in the subarachnoid space in conjunction with the local anesthetic. Additionally, when comparing side effects experienced after surgery, notation should include what anti-emetic medications may have been administered intraoperatively. The variables in our retrospective study varied with each type of anesthetic and between each patient receiving the different types of anesthetics. 

## 5. Conclusions

Based upon our analysis, there is no significant difference between GA and SA in terms of operative time, anesthetic time, and oral pain medications among the patients who received penile implantations. However, with spinal anesthesia, the patients presented with more urticaria. Conversely, greater amounts of intravenous pain medications and treatment of nausea/emesis medication were necessitated by general anesthesia use.
